# Supplementary material for: Engineering the pore environment of antiparallel stacked covalent organic frameworks for capture of iodine pollutants
Source: Nat Commun. 2024 Mar 26;15:2671. doi: 10.1038/s41467-024-46942-0 (PMC10965901; doi:10.1038/s41467-024-46942-0)
Supplement: Supplementary file 1 — Supplementary Information [file 41467_2024_46942_MOESM1_ESM.pdf]

Supplementary Information

Engineering the Pore Environment of Antiparallel Stacked Covalent Organic Frameworks for Capture of Iodine Pollutants

Yinghui Xie,[1] Qiuyu Rong,[1] Fengyi Mao,[1] Shiyu Wang,[1] You Wu,[1] Xiaolu Liu,[1] Mengjie Hao,[1] Zhongshan Chen,[1] Hui Yang,*[,1] Geoffrey I. N. Waterhouse,[2] Shengqian Ma,*[,3] and Xiangke Wang*[,1]

[1] College of Environmental Science and Engineering, North China Electric Power University, Beijing 102206, P.R. China;

[2] School of Chemical Sciences, The University of Auckland, Auckland 1142, New Zealand;

[3] Department of Chemistry, University of North Texas, Denton, Texas 76201, United States

Email: h.yang@ncepu.edu.cn (H.Y.); shengqian.ma@unt.edu (S.M.); xkwang@ncepu.edu.cn (X.W.)

Supplementary Table 1. Fractional atomic coordinates for the antiparallel AA stacking unit cell of ACOF-1

space group P6cc, a = b = 42.31 Å, c = 7.23 Å, α = β= 90°, and γ = 120° Pawley Refinement R_p = 3.89%, R_{wp} = 5.71%			
Atom	x (Å)	y (Å)	z (Å)
C1	0.68885	0.32012	0.14947
C2	0.70294	0.35594	0.14981
C3	0.74077	0.37964	0.1502
C4	0.76391	0.36735	0.17689
C5	0.81223	0.42072	0.14975
C6	0.79169	0.43578	0.12249
C7	0.75616	0.41536	0.12371
C8	0.84965	0.44238	0.15001
C9	0.92754	0.44742	0.28205
C10	0.96458	0.47582	0.27272
C11	0.97623	0.51143	0.27385
C12	0.01114	0.53501	0.27451
C13	0.96211	0.55722	0.26994
N14	0.79801	0.3865	0.17717
N15	0.86928	0.43133	0.21309
N16	0.9038	0.45473	0.20705
O17	0.91976	0.41932	0.3533
O18	0.9514	0.52108	0.28059
H19	0.75426	0.34028	0.20147
H20	0.80367	0.46315	0.09898
H21	0.74075	0.42728	0.10028
H22	0.86011	0.4684	0.09625
H23	0.91193	0.47852	0.15357
H24	0.02074	0.56229	0.2791
H25	0.97602	0.56837	0.14191
H26	0.97806	0.5711	0.38826
C27	0.93846	0.55816	0.27232
H28	0.70587	0.30973	0.14834
H29	0.6269	0.07411	0.12764
C30	0.6422	0.0557	0.32103
H31	0.6184	0.08215	0.37097
C32	0.65558	0.6053	0.69684
H33	0.66425	0.5841	0.90288
H34	0.6288	0.59876	0.91258
H35	0.58917	0.65964	0.08211
H36	0.61607	0.63708	0.14577
H37	0.6294	0.68335	0.23895

Supplementary Table 2. Fractional atomic coordinates for the antiparallel AA stacking unit cell of ACOF-1R

space group P6cc, a = b = 42.14 Å, c = 7.38 Å, α = β = 90°, and γ = 120° Pawley Refinement R_p = 2.48%, R_{wp} = 3.43%			
Atom	x (Å)	y (Å)	z (Å)
C1	0.68722	0.31936	0.22884
C2	0.70127	0.3551	0.22917
C3	0.73901	0.37874	0.22956
C4	0.7621	0.36648	0.25589
C5	0.8103	0.41972	0.22911
C6	0.78981	0.43475	0.20222
C7	0.75437	0.41437	0.20343
C8	0.84763	0.44133	0.22937
C9	0.92534	0.44636	0.35962
C10	0.96229	0.47469	0.35042
C11	0.97391	0.51022	0.35153
C12	0.01111	0.53374	0.35218
C13	0.95983	0.5559	0.34767
N14	0.79612	0.38558	0.25616
N15	0.86722	0.43031	0.29159
N16	0.90166	0.45365	0.28564
O17	0.91758	0.41832	0.4299
O18	0.94914	0.51984	0.35818
H19	0.75247	0.33947	0.28013
H20	0.80176	0.46205	0.17903
H21	0.73899	0.42627	0.18031
H22	0.85807	0.46729	0.17634
H23	0.90977	0.47738	0.23288
H24	0.02069	0.56096	0.35671
H25	0.9737	0.56702	0.22138
H26	0.97574	0.56974	0.46439
C27	0.93623	0.55684	0.35002
H28	0.70419	0.30899	0.22772
H29	0.62541	0.07393	0.2073
C30	0.64068	0.05557	0.39807
H31	0.61693	0.08196	0.44733
C32	0.65402	0.60386	0.76878
H33	0.66267	0.58271	0.97203
H34	0.62731	0.59734	0.9816
H35	0.58777	0.65807	0.16239
H36	0.61461	0.63557	0.22519
H37	0.62791	0.68173	0.3171
C38	0.36359	0.81547	0.74938
O39	0.50131	-0.31282	-0.46184
S40	0.52196	-0.30027	-0.24785
C41	0.56884	-0.27117	-0.2507
F42	0.58513	-0.29118	-0.34289
F43	0.58407	-0.26084	-0.06358
F44	0.57784	-0.23745	-0.35557
O45	0.50015	-0.27651	-0.12828
O46	0.50887	-0.34068	-0.11303
H47	0.33689	0.79826	0.82674
H48	0.35718	0.81943	0.6035
H49	0.38008	0.84332	0.8179

Supplementary Table 3. Summary of the C, N, H, and S contents of ACOF-1 and ACOF-1R

COFs	Theoretical content (wt.%)				Experimental content (wt.%)			
	N(%)	C(%)	H(%)	S(%)	N(%)	C(%)	H(%)	S(%)
ACOF-1	14.87	67.99	5.67		12.29	64.59	5.21	
ACOF-1R	9.41	48.38	4.26	7.17	8.32	44.96	3.81	7.59

Materials characterization

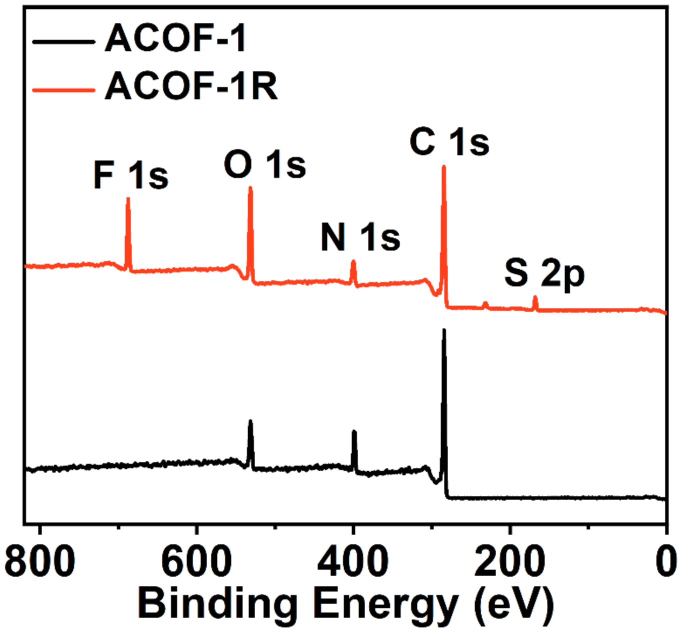

Supplementary Fig. 1 | XPS survey spectra for ACOF-1 and ACOF-1R.

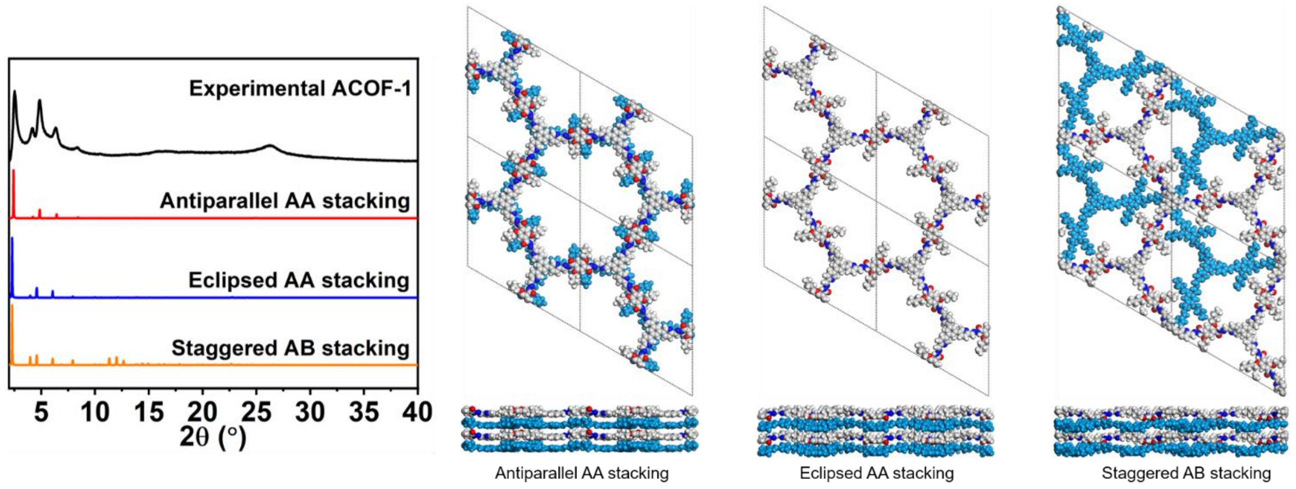

Supplementary Fig. 2 | Experimental PXRD pattern of ACOF-1 with the possible structural models of ACOF-1 assuming the antiparallel AA, eclipsed AA, and staggered AB stacking configurations.

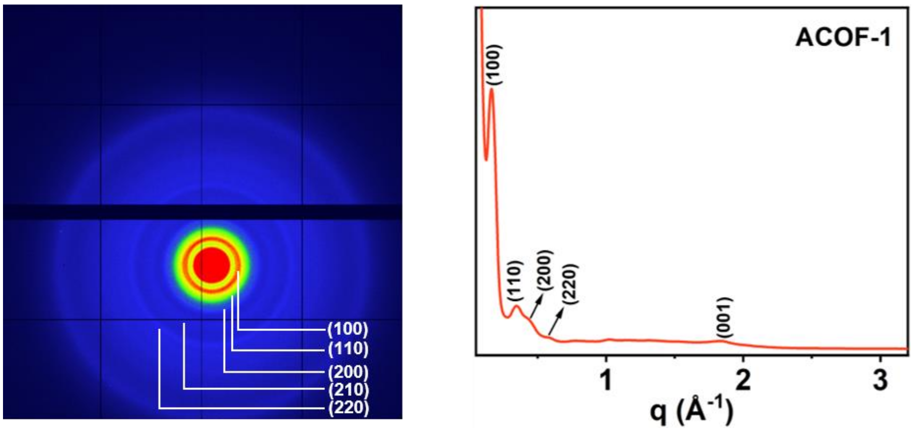

Supplementary Fig. 3 | 2D WAXS image (left) and pattern (right) of ACOF-1.

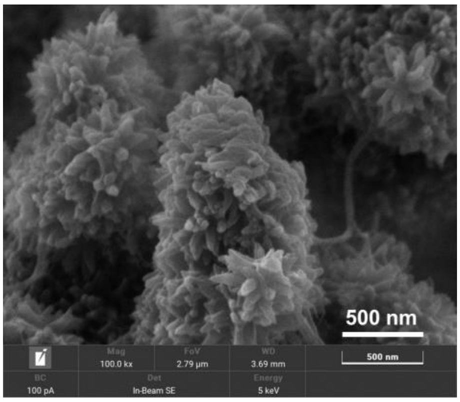

Supplementary Fig. 4 | SEM image of ACOF-1.

Total charge density and interlayer differential charge density calculations

Density functional theory (DFT) calculations[1, 2] were carried out in the Vienna *ab initio* simulation package (VASP) based on the plane-wave basis sets with the projector augmented-wave method.[3, 4] The exchange-correlation potential was treated by using a generalized gradient approximation (GGA) with the Perdew-Burke-Ernzerhof (PBE) parametrization.[5] Van der Waals interactions were considered at the vdW-DF level with the nonlocal vdW-DF2 functional.[6] The energy cutoff was set to be 500 eV. The Brillouin-zone integration was sampled with a Γ-centered Monkhorst-Pack mesh of 1 × 1 × 3 (1 × 1 × 1) for antiparallel and eclipsed cells, respectively.[7] The structures were fully relaxed until the maximum force on each atom was less than 0.01 eV/Å, and the energy convergent standard was 10^{-5} eV.

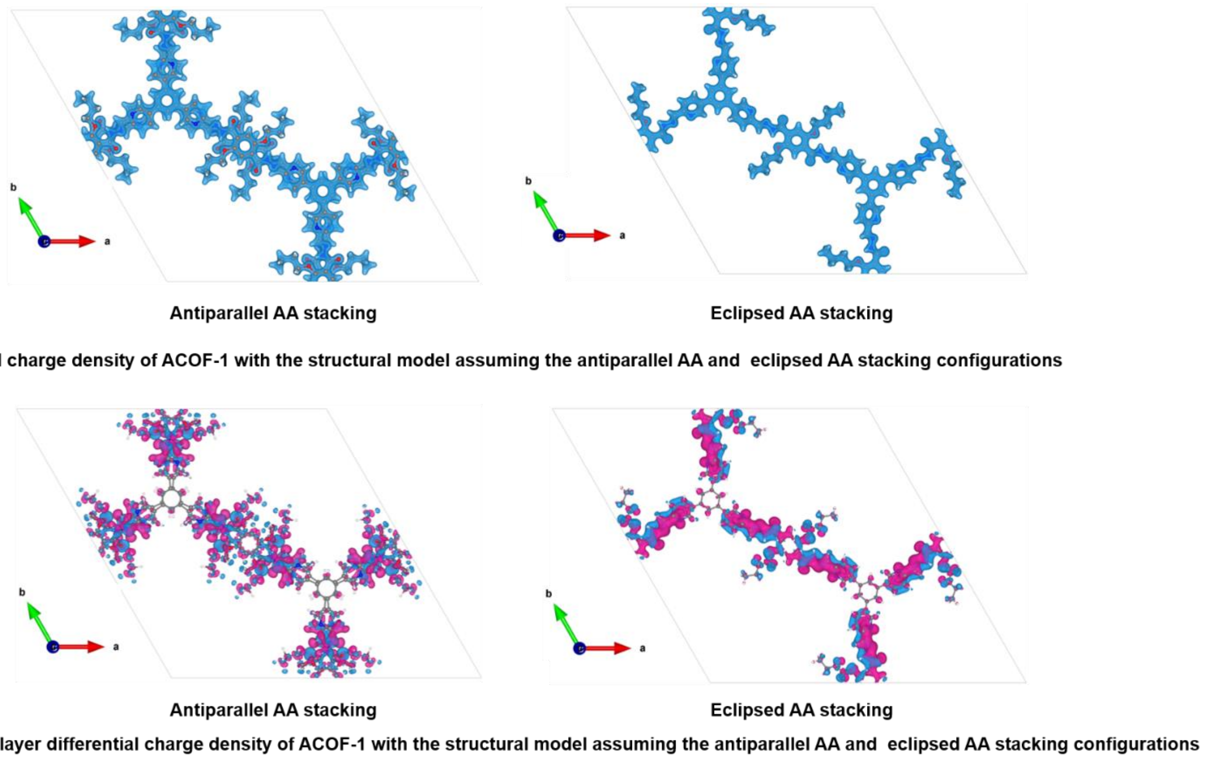

Supplementary Fig. 5 | Total charge density of ACOF-1 with the structural model assuming the antiparallel AA and eclipsed AA stacking configurations in an iso-surface of 0.13 e per Å^3 (top). Interlayer differential charge density of ACOF-1 with the structural model assuming the antiparallel AA and eclipsed AA stacking configurations in an isosurface of 5.7×10^{-4} e per Å^3 (bottom). Pink and blue regions represent decreased or increased charge density, respectively.

Supplementary Table 4. Total energy of DFT-optimized ACOF-1 in the antiparallel AA and eclipsed AA unit cell.

Stacking model	Interlayer π-π distance (Å)	Total energy (eV)
Antiparallel AA	3.62	-2489.21
Eclipsed AA	3.96	-2487.43

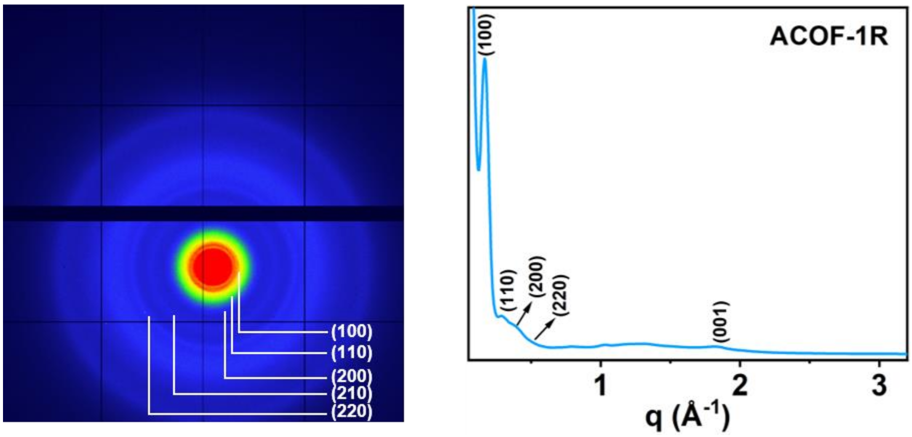

Supplementary Fig. 6 | 2D WAXS image (left) and pattern (right) of ACOF-1R.

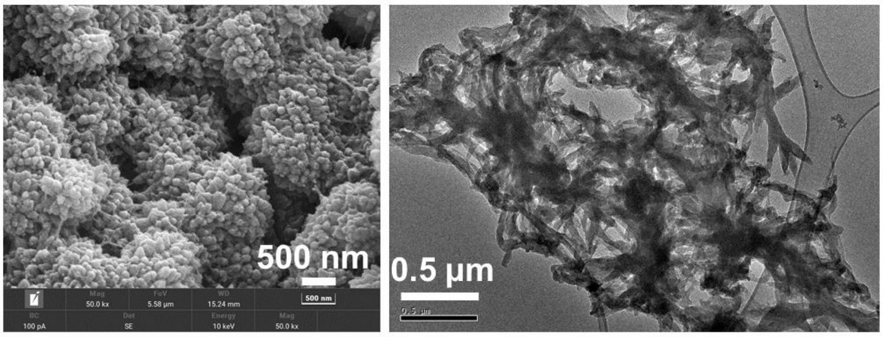

Supplementary Fig. 7 | SEM (left) and TEM (right) images of ACOF-1R.

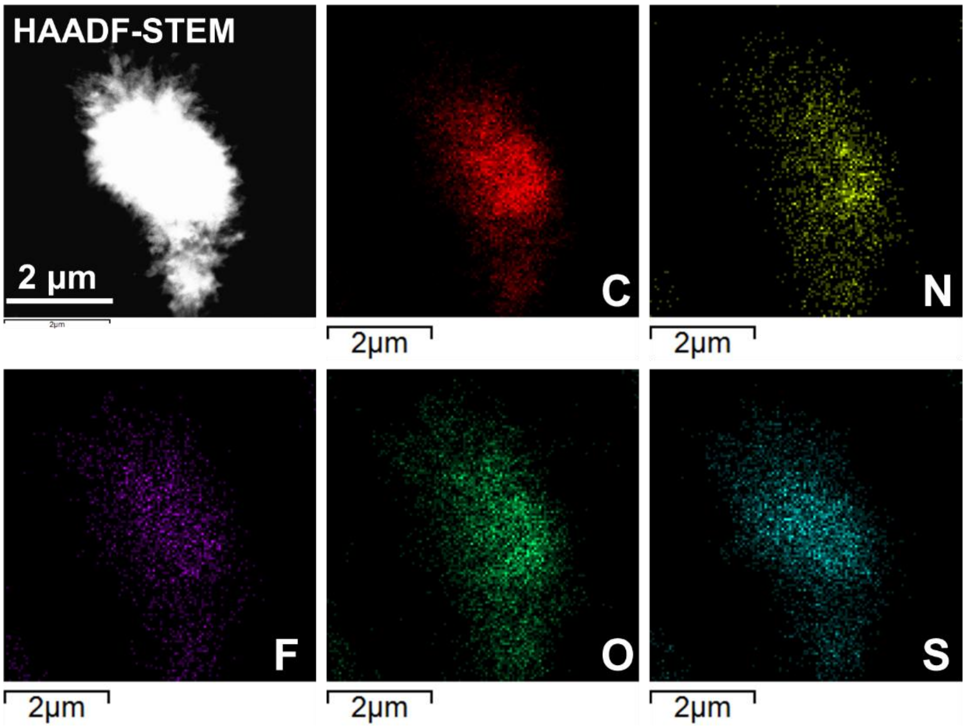

Supplementary Fig. 8 | HAADF-STEM and mapping images of ACOF-1R.

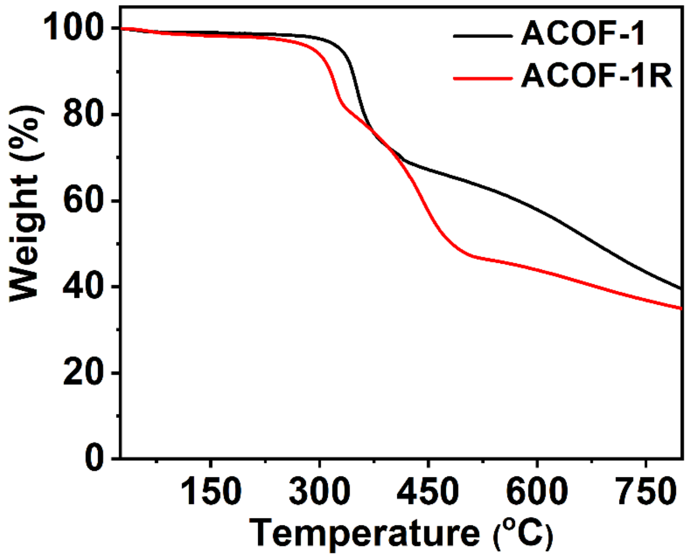

Supplementary Fig. 9 | TGA analysis of ACOF-1 and ACOF-1R. Samples were heated at 10 °C/min in N_2.

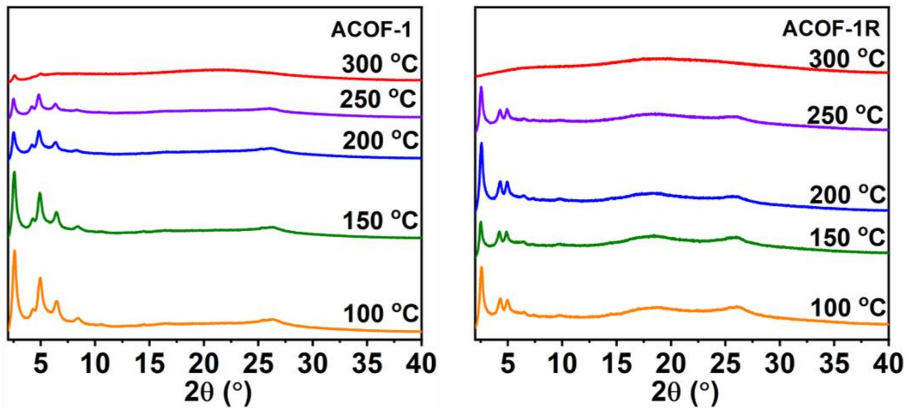

Supplementary Fig. 10 | PXRD patterns of ACOF-1 and ACOF-1R after heating at different temperatures for 12 h (under vacuum).

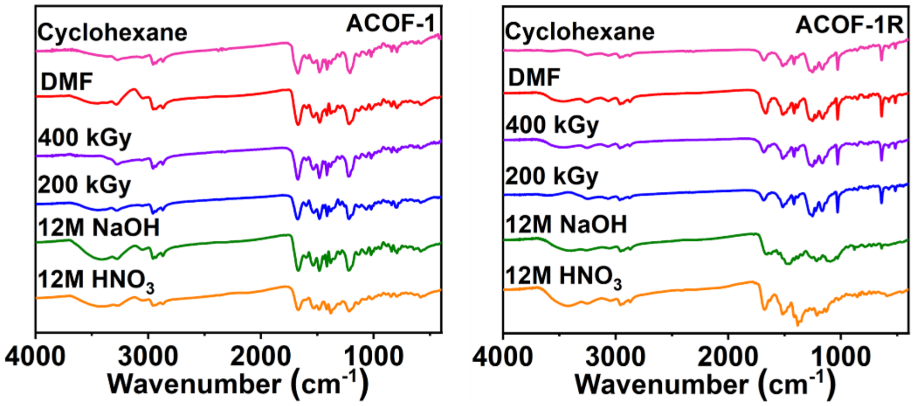

Supplementary Fig. 11 | FT-IR spectra for ACOF-1 and ACOF-1R after different treatments.

Supplementary Methods

Static I_2 and CH_3I vapor capture experiments.

Static I_2 and CH_3I uptake experiments based on gravimetric measurements were performed using the following method. Three glass vials were placed in a teflon container (see below Supplementary Fig. 12): The first vial contained ACOF-1 (10 mg), the second vial containing iodine (1 g) or methyl iodide (1 mL), the third vial was empty and used as a reference for calculating the I_2 and CH_3I uptake by the vial. The teflon container was then sealed and transferred into an oven operating at 75 °C. At regular intervals, the teflon container was removed from the oven and cooled to room temperature. The COF sample vial and the empty vial were weighted and then returned to the oven for further I_2 or CH_3I adsorption. These procedures were repeated until the vial containing ACOF-1 achieved a constant weight (indicating adsorption equilibrium was reached).

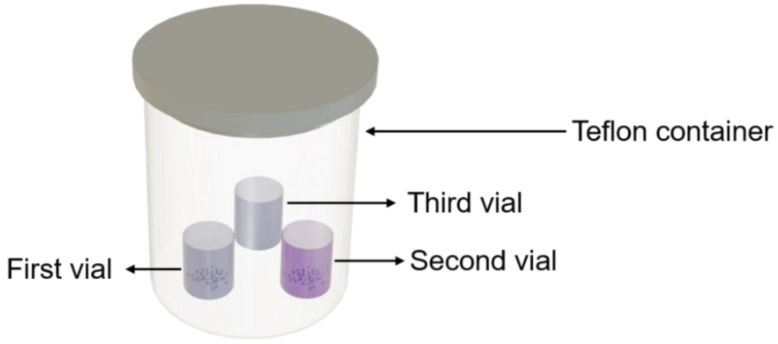

Supplementary Fig. 12 | Schematic illustration of the apparatus used in static I_2 and CH_3I vapor uptake experiments. First vial: contained the COF sample, second vial contained I_2/or CH_3I, third vial: reference (blank vial).

The static I_2 and CH_3I vapor uptake capacities (q_t, g/g) of ACOF-1 at regular intervals were calculated using the following equation:

$$q_t=\frac{(m_t-m_1)-(m_{r,t}-m_{r,0})}{m_1-m_0}$$

where q_t (g/g) is the static I_2 vapor (or CH_3I) uptake capacity at time t, m_t (g) is the weight of first vial containing the COF sample at time t, m_1 (g) is the weight of the first vial with COF sample before the adsorption experiment, m_0 (g) is the weight of first vial without COF sample, $m_{r,t}$ is the weight of third vial (reference) at time t, $m_{r,0}$ (g) is the weight of the third vial before adsorption experiment.

Dynamic iodine vapor capture studies.

The I_2 capture breakthrough experiment was conducted using a lab-scale fixed-bed reactor at room temperature (25 °C) and 75 °C. In a typical experiment, 50.0 mg of ACOF-1 was packed into a quartz column (6.0 mm inside diameter, 1 mm thickness, 400 mm length) with degreased cotton filling the void space. Then, a dry nitrogen gas flow (5 mL/min) was passed through a glass bottle containing I_2, with the resulting I_2/N_2 flow passed through the adsorbent column. The flow rate of I_2 was measured to be around 100 mg/h. The effluent from the adsorbent column was passed through cyclohexane for scrubbing the I_2 vapor, with the I_2 content in the cyclohexane then detected by UV-Vis spectroscopy at a wavelength of 523 nm. Blank was determined by using degreased cotton (without the COF samples) at 25 °C. The final dynamic adsorption capacity of iodine amounts was determined by the gravimetric method.

Dynamic methyl iodide vapor capture studies.

For the CH_3I capture breakthrough experiment, a dry nitrogen flow (5 mL/min) was bubbled through CH_3I in a pressure-resistant quartz bottle, then the resulting CH_3I/N_2 flow passed through the adsorbent column. The flow rate of CH_3I was measured to be around 3.97 mg/min. The effluent from the adsorbent column was analyzed by gas chromatography (Shimadzu GC2030) equipped with a flame ionization detector and GC column (SH-1, 0.25 mm × 0.25 μm × 30 m). N_2 was used as a carrier gas. Blank was determined by using degreased cotton (without the COF samples) at 25 °C. The final dynamic adsorption capacity of methyl iodide amounts was determined by the gravimetric method.

Triiodide adsorption studies.

2.0 mg of ACOF-1R was added into an I_2/NaI solution (I_2: 50 ppm, NaI: 100 ppm, 10 mL) and the resulting dispersion was continuously shaken at room temperature. Ten parallel experiments were carried out to determine the adsorption kinetics of ACOF-1R. Vials were removed one by one from the shaker at specific intervals. After filtration, the I_2 (trace amount) and I_3^- concentrations in the filtrate were determined by UV-Vis spectroscopy at 461 nm and 288 nm, respectively. The I_2/I_3^- adsorption experiments were carried out under similar conditions, only changing the I_2/NaI solution to an iodine-saturated solution.

The efficiency of triiodide removal (%) by the adsorbent COFs subject to study was determined using the following equation:

$$E(\%)=\frac{C_0-C_t}{C_0}\times 100\%$$

where C_0 (mg/L) and C_t (mg/L) are the concentrations of triiodide before and after adsorption, respectively. The amount of adsorbed triiodide was determined using the following equation:

$$q_t=\frac{(C_0-C_t)\times V}{m}$$

where q_t (mg/g) is the amount of triiodide adsorbed per gram of adsorbent at time t (min), C_0 (mg/L) and C_t (mg/L) are the initial and residual concentrations of triiodide in the stock solution and filtrate, respectively, m (g) is the mass of adsorbent used in the study.

Selectivity of triiodide ion capture

The effect of possible competing anions on triiodide adsorption by ACOF-1R was studied by adding NO_3^-, HCO_3^-, Cl^-, CH_3COO^-(Ac^-), or SO_4^{2-}, (concentration of 100 ppm) into an I_2/NaI solution (I_2: 50 ppm, NaI: 100 ppm) solution. ACOF-1R (2.0 mg) was added to 10 mL of I_2/NaI solutions containing NO_3^-, HCO_3^-, Cl^-, CH_3COO^- (Ac^-), or SO_4^{2-}, respectively. After shaking at a rate of 180 rpm for 24 h, the adsorbent was removed on a 0.22 μm membrane filter, and residual triiodide concentrations were quantified by UV-Vis spectroscopy.

Reusability of ACOF-1R

After I_3^- adsorption experiments, ACOF-1R was immersed in ethanol (30 mL). The solvent was exchanged every 1 h until the solution remained colorless. The solid COF was then dispersed in 40 mL of saturated NaCl overnight. The solid

COF was then filtered and washed several times with deionized water. Then, the ACOF-1R was subsequently returned to an I_3^- solution for further adsorption tests.

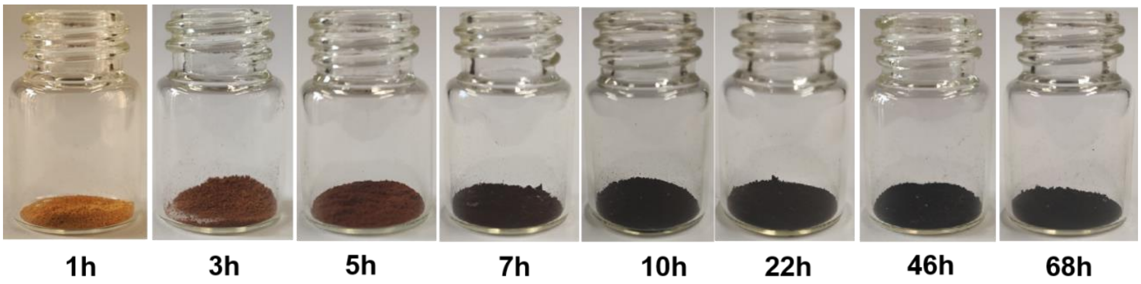

Supplementary Fig. 13 | Photographs of ACOF-1 during the adsorption of iodine vapor.

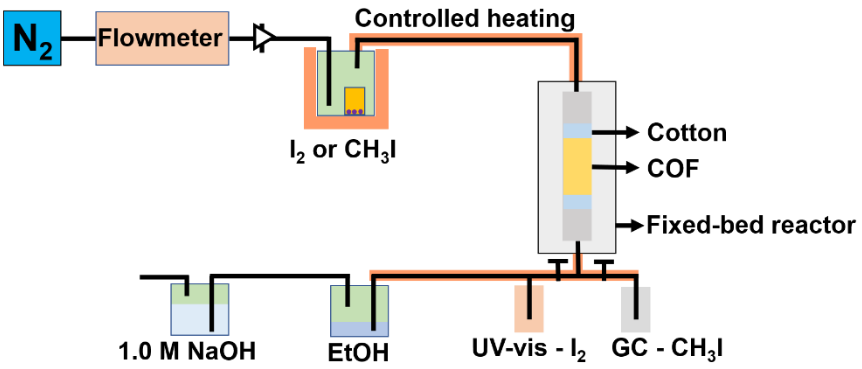

Supplementary Fig. 14 | Schematic illustration of the apparatus used in I_2 and CH_3I breakthrough experiments. The fixed bed column was packed with ACOF-1, with degreased cotton used to fill the void space.

Supplementary Table 5. Comparison of the static I_2 vapor capture capacities of ACOF-1 with other adsorbents

Type	Adsorbent	T (°C)	S_{BET}	Pore volume (cm^3/g)	I_2 uptake (g/g)	$T_{80\%}$ (h)	$K_{80\%}$ (g/g/h)	Ref.
POPs	PSIF-5a	75	574	1.41	3.01	5	0.48	8
	SCMP-2	80	308	1.50	2.22	3	0.59	9
	ImCMP-1	80	356.9	0.33	2.36	5	0.37	10
	BTPOC	75	605		3.21	10	0.26	11
	T_COP-1	75	206	0.22	4.86	12	0.32	12
	CMP-4	75	9.5		2.08			13
	MeBID[3]	75	7.8		5.12			14
	CTF-CTTD-500	75	1334	1.40	3.87	21	0.18	15
	PAF-24	75	136		2.76			16
	P-PON	75	873		2.50	8	0.25	17
MOFs	AlOC-27-NC	80	285		0.50	2.5	0.20	18
	IL@PCN-333(Al)	75	1635	1.40	7.35	3	2.45	19
	MOF-808	80	1930	0.82	2.18	13	0.17	20
	UiO-66-NH-T.D	75	317	0.20	1.33	2.5	0.53	21
	MFM-300(Sc)	70	1250	0.5	1.54	10	0.19	22
COFs	USTB-1	75	1322	0.66	4.45	80	0.22	23
	USTB-1c	75	1454	1.01	5.80	80	0.28	23
	JUC-561	75	2359	1.92	8.19	18	0.70	24
	TAPA-PDA COF	77	685	0.73	5.09	10	0.51	25
	HDOBD	77	14	0.05	4.47	12	0.37	26
	P-COF	77	1056	0.6	6.19	2	2.50	27
	$Cu_{0.25}$Pc-COF	80	9.3		2.99	8	0.30	28
	BTT-TAPT-COF	78	864	0.56	2.76	5	0.44	29
	TFB−DB COF	75	734.2	0.11	6.40	15	0.37	30
	COF-TAPT	75	2348	0.97	8.61	18	0.48	31
	COF-TAPB	75	2290	0.89	7.94	24	0.33	31
	SIOC-COF-7	75	618	0.41	4.81	15	0.32	32
	FAL-COF-1	75	168	0.32	5.49			33
	COF-PA	77	2174		4.47	16	1.30	34
	iCOF-AB-50	75	1390	1.21	10.21	7.25	1.12	35
	Meso-COF-3	75	982	0.84	4.00	75		36
	QTD-COF-V	75			6.29	2.5	2.51	37
	SCU-COF-2	75	413.4	0.46	6.0	24	0.25	38
	TPB-DMTP COF	77	1927	1.28	6.26	36	0.17	39
	TTA-TTB COF	77	1733	1.01	4.95	96	0.14	39
	ACOF-1	75	1698	1.04	4.73	22	0.20	This work

Supplementary Table 6. Comparison of the static CH_3I vapor capture capacities of ACOF-1 with other adsorbents

Type	Adsorbent	T (°C)	CH_3I uptake (g/g)	Time (h)	Adsorption mechanisms	Ref.
POPs	MHP-P5Q	75	0.80	1	Methylation reaction	[40]
POPs	PHCP@PES	75	0.19	1		[41]
MOFs	ECUT-300-25-Me	75	2.03	12	Methylation reaction/ hydrogen bonding/ coordination interaction	[42]
MOFs	ECUT-300-25-Ac	75	2.46	12	Methylation reaction/ hydrogen bonding/ coordination interaction	[42]
MOFs	MIL-101-Cr-HMTA	75	1.35		Methylation reaction	[43]
MOFs	MIL-53	35	0.13		Hydrogen bonding	[44]
MOFs	MIL-120	35	0.16		Hydrogen bonding	[44]
MOFs	HKUST-1	35	0.43		Coordination interaction	[44]
MOFs	MIL-101-RSO_3Ag	30	0.16		Coordination interaction	[45]
COFs	TPB-DMTP-COF	75	0.70		Coordination interaction	[39]
COFs	COF-OH-0	75	1.40			[35]
COFs	TFPA-TAPT	75	1.37		Methylation reaction	[31]
COFs	COF-TAPT	75	1.53	120	Methylation reaction	[31]
COFs	COF-TAPB	75	0.81	120	Methylation reaction	[31]
COFs	SCU-COF-2	75	1.45	96	Methylation reaction	[38]
COFs	ACOF-1	75	1.61	24	Methylation reaction/ coordination interaction	This work

Supplementary Table 7. Comparison of the dynamic I_2 vapor capture capacities of ACOF-1 with other adsorbents

Type	Adsorbent	T(°C)	Pressure	I_2 uptake (g/g)	Ref.
Zeolite	HISL	R.T.	Ambient	0.53	[46]
	SL-1	R.T.	Ambient	0.48	[46]
	Si-BEA	R.T.	Ambient	0.47	[46]
	Ag^0@MOR	R.T.	Ambient	0.08	[43]
Inorganic material	AC	R.T.	Ambient	0.70	[46]
	Zn-Sacc	R.T.	Ambient	0.05	[46]
	SCU-SnS	75	Ambient	1.66	[47]
MOFs	ZIF-8	R.T.	Ambient	0.03	[46]
	HKUST-1	R.T.	Ambient	0.38	[46]
	MIL-101-TED	150	Ambient	0.43[a]	[43]
	MIL-101-Cr-HMTA	150	Ambient	0.41[a]	[43]
COFs	SCU-COF-2	R.T.	Ambient	0.98	[38]
	COF-OH-50	R.T	Ambient	1.70	[35]
	iCOF-AB-50	R.T	Ambient	2.79	[35]
	COF-TAPT	25	Ambient	2.38	[31]
	COF-TAPB	25	Ambient	2.18	[31]
	TGDM	150	Ambient	0.30[a]	[48]
	ACOF-1	25	Ambient	2.16	This work

[a] Measurements were taken at a concentration of 150 ppm of I_2, R.T. = room temperature

Supplementary Table 8. Comparison of the dynamic CH_3I vapor capture capacities of ACOF-1 with other adsorbents

Type	Adsorbent	T(°C)	Pressure	CH_3I uptake (g/g)	Ref.
Zeolite	HISL	30	Ambient	0.42	[46]
	Ag^+@13X	30	Ambient	0.45	[43]
	Ag^+@MOR	30	Ambient	0.29	[43]
	Ag^+@ZSM5	30	Ambient	0.28	[43]
	Ag^0@MOR	30	Ambient	0.25	[43]
Composites	TED-AC	30	Ambient	0.52	[43]
	HMTA-AC	30	Ambient	0.54	[43]
MOFs	MIL-101-Cr-TED	30	Ambient	1.60	[43]
	MIL-101-Cr-HMTA	25	Ambient	1.66	[43]
	ECUT-300-200Ac	25	Ambient	2.80	[42]
	ECUT-300-200Ac	150	Ambient	0.87	[42]
COFs	SCU-COF-2	25	Ambient	0.564	[38]
	iCOF-AB-50	25	Ambient	0.62	[35]
	COF-TAPT	25	Ambient	1.30	[31]
	COF-TAPB	25	Ambient	0.71	[31]
	ACOF-1	25	Ambient	0.74	This work

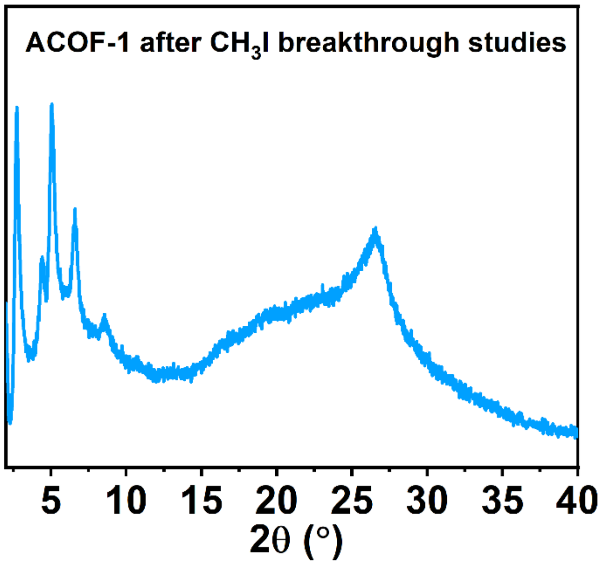

Supplementary Fig. 15 | PXRD of ACOF-1 after the CH_3I breakthrough experiment.

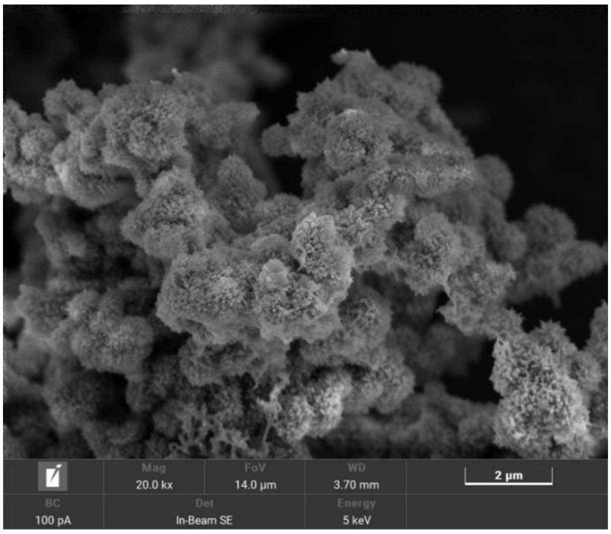

Supplementary Fig. 16 | SEM image of ACOF-1 after the CH_3I breakthrough experiment.

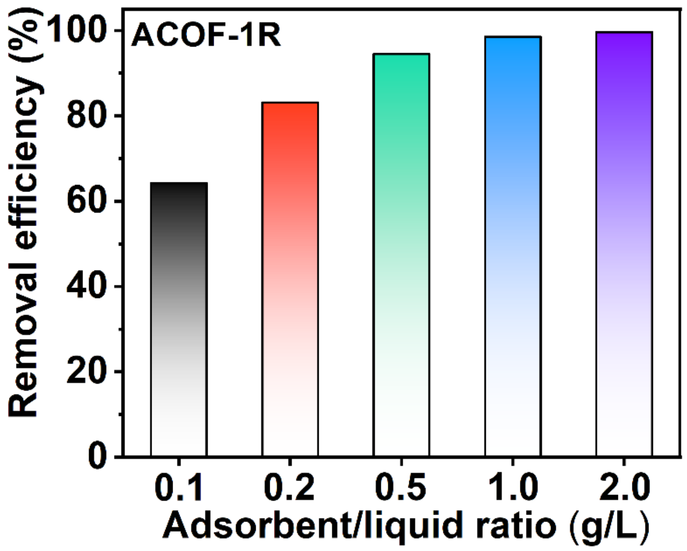

Supplementary Fig. 17 | I_2/I_3^- removal by ACOF-1R at various adsorbent/liquid ratios.

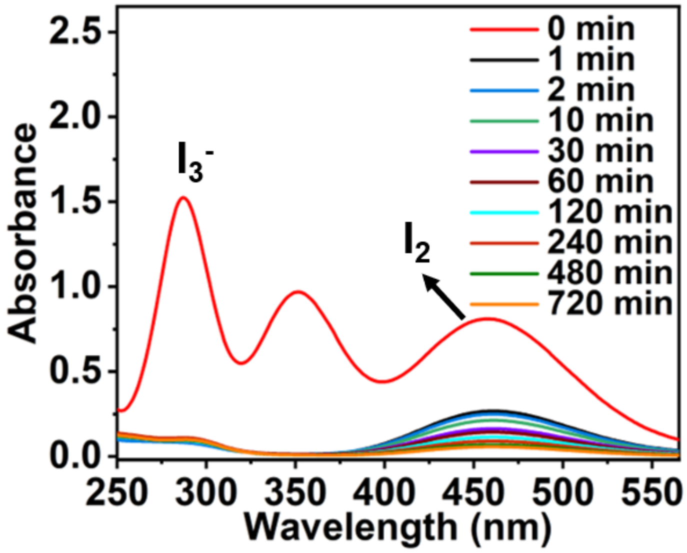

Supplementary Fig. 18 | I_2/I_3^- removal by ACOF-1R in a saturated aqueous iodine solution.

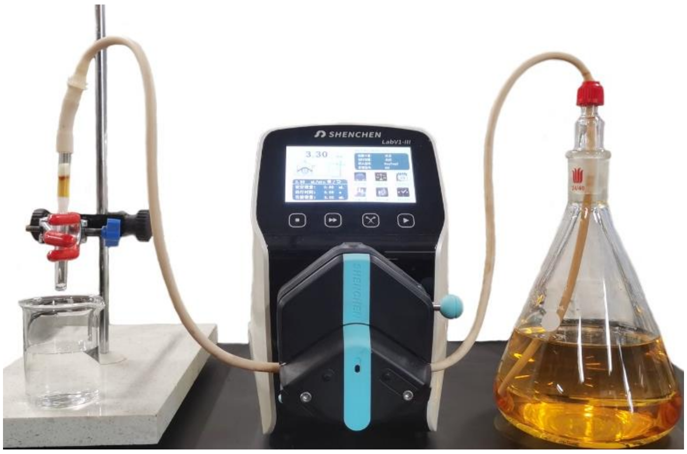

Supplementary Fig. 19 | Experimental set-up used for I_3^- removal from groundwater by ACOF-1R.

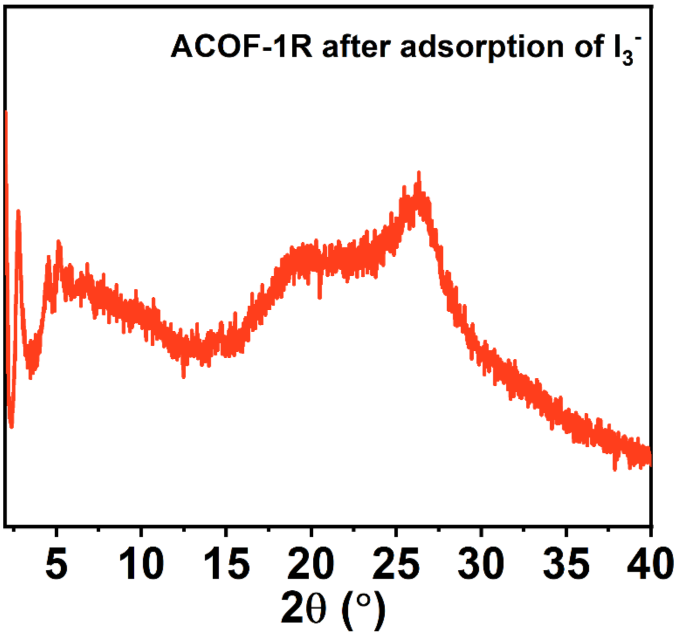

Supplementary Fig. 20 | PXRD of ACOF-1R after adsorption of I_3^-.

Supplementary Table 9. Comparison of the iodine/iodine ions (I_3^-) adsorption capacities of ACOF-1R with other adsorbents

Type	Adsorbent	S_{BET}	I_2/I_3^- uptake (g/g)	Conditions	Mechanism	Recycle	Ref.
POPs	C[4]P-BTP	20.5	3.24	15 mg/mL I_2 + 30 mg/mL KI	Chemisorption	Heat	[49]
	C[4]P-BT	28.3	2.32				
	C[4]P-TTP	19	2.51				
	C[4]P-BP	41.9	2.37				
	C[4]P-TPE	77.7	2.99				
	C[4]P-DPP	110.0	1.58				
POCs	HPOC-101	373	1.38	0.337 mM KI/I_2	Physicochemical adsorption	MeOH	[50]
COPs	CaCOP1	10.86	2.40	1000 ppm KI/I_2	Physicochemical adsorption	EtOH	[51]
	CaCOP2	20.16	2.81				
	CaCOP3	81.09	3.10				
CMPs	CMP-4	9.5	94%	254 ppm I_2	Chemisorption	MeOH	[13]
POPs	C-poly-1_5	96.16	3.2	1.18 mM I_2	Physical adsorption	MeOH	[52]
MFPs	MFP	1440	~99%	1500 ppm KI/I_2	Chemisorption		[53]
AlMCs	AlMC-1	233	1.03	100000 ppm KI/I_2	Ion-exchange	EtOH	[54]
HOFs	H_COF-1		2.1	25 mM I_2 + 75 mM KI	Physicochemical adsorption	DMSO	[55]
	H_COF-7		1.39	0.87 M I_2 + 1.02 M KI	Ion-exchange	DMSO	[56]
Xerogels	G-TP5		0.25	254 ppm I_2	Physical adsorption	MeOH	[57]
	δ-Bi_2O_3@PES	10.96	0.095	500 ppm I^-/IO_3^-	Chemisorption	NaCl	[58]
COFs	TAPB-BPDA	1082	0.988	180 ppm I_2	Chemisorption	MeOH	[59]
	ACOF-1R	422.3	4.46	I_2: 50 ppm, NaI: 100 ppm	Ion-exchange together with physicochemical adsorption	EtOH/NaCl	This work

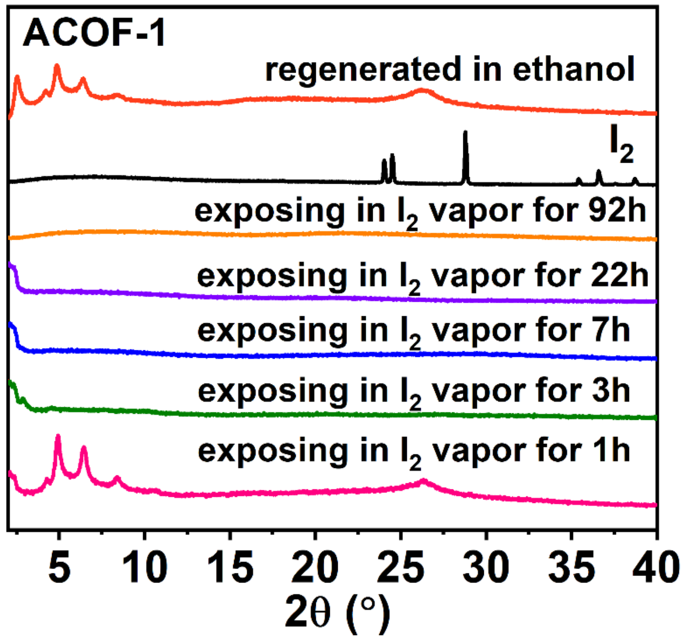

Supplementary Fig. 21 | PXRD patterns of ACOF-1 after different I_2 exposure times, then after regeneration of the ACOF-1 in ethanol.

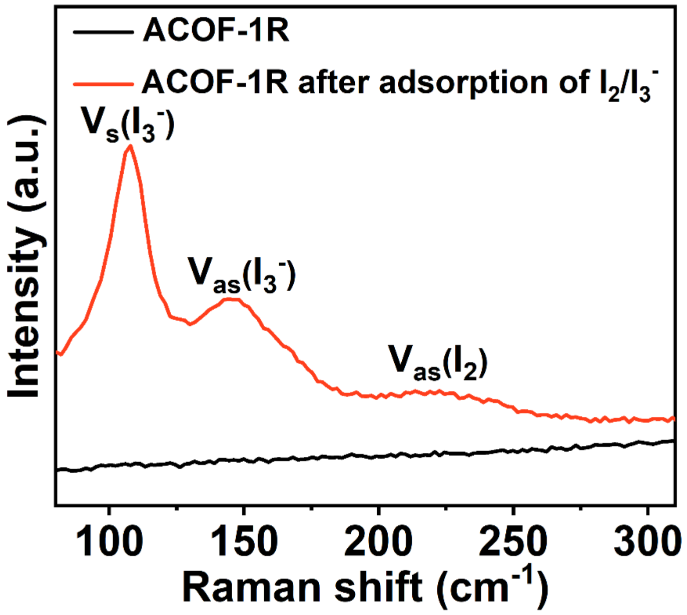

Supplementary Fig. 22 | Raman spectra of ACOF-1R before and after adsorption of I_2/I_3^-.

Density functional theory (DFT) calculations

A fragment of ACOF-1, ACOF-1R ($CF_3SO_3^-$), I_2, I_3^-, I_5^- and the structures for adsorbed iodine species on COFs were all optimized under the framework of the density of functional theory (DFT) with B3LYP functional[60-62], using a DFT-D3 dispersion correction method and basis set of 6-31g(d)+SDD[63-65]. Here, the SDD effective core potential was used to describe the atomic orbitals and relativistic effects of iodine atoms. The reactants, products, and transition state of the methylation reaction of pyridine were also calculated as above. Vibrational frequency analyses were carried out for the optimized structures using the same calculation method. In order to describe solvation effects, the SMD (Solvation Model Based on Density)[66] implicit solvent model was used in the reaction calculations. The thermodynamic correction terms and Gibbs free energy of the structures at 298.15K were then obtained using the Shermo program[67]. In order to obtain the electron energy with higher accuracy, single point calculations for the optimized structures with B3LYP functional and 6-311+G(d,p) & SDD basis set were performed. Finally, the single point energy was added to the free energy correction calculated before obtaining the Gibbs free energy. All these DFT calculations above were performed using Gaussian 16 program suite[68].

The adsorption energy of I_2/hydrazine sites, I_2/pyridine sites, I_3^-/pyridine sites, and I_5^-/pyridine sites were calculated from the formula:

$$E(adsorb)=E(A+B)-E(A)-E(B)$$

where E(A) and E(B) are the free energy of isolated molecules, E(A+B) is the total energy of the adsorption complex structures.

The electrostatic surface potentials (ESP) were calculated using the Multiwfn program[69, 70] and then rendered using the Gauss View 6.0.

Supplementary References

1. Hohenberg, P. & Kohn, W. Inhomogeneous electron gas. *Phys. Rev.* **136**, B864-B871 (1964).
2. Kohn, W. & Sham, L. J. Self-consistent equations including exchange and correlation effects. *Phys. Rev.* **140**, A1133-A1138 (1965).
3. Blöchl, P. E. Projector augmented-wave method. *Phys. Rev. B* **50**, 17953-17979 (1994).
4. Kresse, G. & Furthmüller, J. Efficient iterative schemes for ab initio total-energy calculations using a plane-wave basis set. *Phys. Rev. B* **54**, 11169-11186 (1996).
5. Perdew, J. P., Burke, K. & Ernzerhof, M. Generalized gradient approximation made simple. *Phys. Rev. Lett.* **77**, 3865-3868 (1996).
6. Lee, K., Murray, É. D., Kong, L., Lundqvist, B. I. & Langreth, D. C. Higher-accuracy van der Waals density functional. *Phys. Rev. B* **82**, 081101 (2010).
7. Monkhorst, H. J. & Pack, J. D. Special points for Brillouin-zone integrations. *Phys. Rev. B* **13**, 5188-5192 (1976).
8. Janeta, M., Bury, W. & Szafert, S. Porous silsesquioxane-imine frameworks as highly efficient adsorbents for cooperative iodine capture. *ACS Appl. Mater. Interfaces* **10**, 19964-19973 (2018).
9. Qian, X., Zhu, Z.-Q., Sun, H.-X., Ren, F., Mu, P., Liang, W., Chen, L. & Li, A. Capture and reversible storage of volatile iodine by novel conjugated microporous polymers containing thiophene units. *ACS Appl. Mater. Interfaces* **8**, 21063-21069 (2016).
10. Meng, X., Liu, Y., Wang, S., Ye, Y., Song, X. & Liang, Z. Molecule-guided synthesis of conjugated microporous polymers with imidazole derivative units for efficient capture of volatile iodine. *Micropor. Mesopor. Mater.* **336**, 111871 (2022).
11. Liu, C., Li, W., Liu, Y., Wang, H., Yu, B., Bao, Z. & Jiang, J. Porous organic cages for efficient gas selective separation and iodine capture. *Chem. Eng. J.* **428**, 131129 (2022).
12. Hassan, A., Alam, A., Ansari, M. & Das, N. Hydroxy functionalized triptycene based covalent organic polymers for ultra-high radioactive iodine uptake. *Chem. Eng. J.* **427**, 130950 (2022).
13. Dai, D., Yang, J., Zou, Y.-C., Wu, J.-R., Tan, L.-L., Wang, Y., Li, B., Lu, T., Wang, B. & Yang, Y.-W. Macrocyclic arenes-based conjugated macrocycle polymers for highly selective CO_2 capture and iodine adsorption. *Angew. Chem. Int. Ed.* **60**, 8967-8975 (2021).
14. Yu, X., Wu, W., Zhou, D., Su, D., Zhong, Z. & Yang, C. Bisindole [3]arenes-indolyl macrocyclic arenes having significant iodine capture capacity. *CCS Chem.* **4**, 1806-1814 (2022).
15. Jiang, Q., Huang, H., Tang, Y., Zhang, Y. & Zhong, C. Highly porous covalent triazine frameworks for reversible iodine capture and efficient removal of dye. *Ind. Eng. Chem. Res.* **57**, 15114-15121 (2018).

16. Yan, Z., Yuan, Y., Tian, Y., Zhang, D. & Zhu, G. Highly efficient enrichment of volatile iodine by charged porous aromatic frameworks with three sorption sites. *Angew. Chem.Int. Ed.* **54**, 12733-12737 (2015).

17. Ahmad, I., Noh, H.-J., Yu, S.-Y., Jeon, J.-P., Sun, Q., Mahmood, J. & Baek, J.-B. 3D porous fused aromatic networks for high performance gas and iodine uptakes. *Adv. Mater. Interfaces* **8**, 2101373 (2021).

18. Yao, S., Fang, W.-H., Sun, Y. & Wang, S.-T. Zhang, J. Mesoporous assembly of aluminum molecular rings for iodine capture. *J. Am. Chem. Soc.* **143**, 2325-2330 (2021).

19. Tang, Y., Huang, H., Li, J., Xue, W. & Zhong, C. IL-induced formation of dynamic complex iodide anions in IL@MOF composites for efficient iodine capture. *J. Mater. Chem. A* **7**, 18324-18329 (2019).

20. Chen, P., He, X., Pang, M., Dong, X., Zhao, S. & Zhang, W. Iodine capture using Zr-based metal-organic frameworks (Zr-MOFs): Adsorption performance and mechanism. *ACS Appl. Mater. Interfaces* **12**, 20429-20439 (2020).

21. Zahid, M., Zhang, D., Xu, X., Pan, M., ul Haq, M. H., Reda, A. T. & Xu, W. Barbituric and thiobarbituric acid-based UiO-66-NH_2 adsorbents for iodine gas capture: Characterization, efficiency and mechanisms. *J. Hazard. Mater.* **416**, 125835 (2021).

22. Zhang, X., da Silva, I., Godfrey, H. G. W., Callear, S. K., Sapchenko, S. A., Cheng, Y., Vitorica-Yrezabal, I., Frogley, M. D., Cinque, G., Tang, C. C., Giacobbe, C., Dejoie, C., Rudic, S., Ramirez-Cuesta, A. J., Denecke, M. A., Yang, S. & Schroder, M. Confinement of iodine molecules into triple-helical chains within robust metal-organic frameworks. *J. Am. Chem. Soc.* **139**, 16289-16296 (2017).

23. Liu, C., Jin, Y., Yu, Z., Gong, L., Wang, H., Yu, B., Zhang, W. & Jiang, J. Transformation of porous organic cages and covalent organic frameworks with efficient iodine vapor capture performance. *J. Am. Chem. Soc.* **144**, 12390-12399 (2022).

24. Chang, J., Li, H., Zhao, J., Guan, X., Li, C., Yu, G., Valtchev, V., Yan, Y., Qiu, S. & Fang, Q., Tetrathiafulvalene-based covalent organic frameworks for ultrahigh iodine capture. *Chem. Sci.* **12**, 8452-8457 (2021).

25. Chen, R., Hu, T., Zhang, W., He, C. & Li, Y. Synthesis of nitrogen-containing covalent organic framework with reversible iodine capture capability. *Micropor. Mesopor. Mater.* **312**, 110739 (2021).

26. Geng, T.-M., Wang, F.-Q., Fang, X.-C. & Xu, H. Dual functional N,O,P containing covalent organic frameworks for adsorping iodine and fluorescence sensing to p-nitrophenol and iodine. *Micropor. Mesopor. Mater.* **317**, 111001 (2021).

27. Li, Y., Li, X., Li, J., liu, W., Cheng, G. & Ke, H. Phosphine-based covalent organic framework for highly efficient iodine capture. *Micropor. Mesopor. Mater.* **325**, 111351 (2021).

28. Liu, X., Zhang, A., Ma, R., Wu, B., Wen, T., Ai, Y., Sun, M., Jin, J., Wang, S. & Wang, X. Experimental and theoretical insights into copper phthalocyanine-based covalent organic frameworks for highly efficient radioactive iodine capture. *Chin. Chem. Lett.* **33**, 3549-3555 (2022).

29. Pan, X., Qin, X., Zhang, Q., Ge, Y., Ke, H. & Cheng, G. N- and S-rich covalent organic framework for highly efficient removal of indigo carmine and reversible iodine capture. *Micropor. Mesopor. Mater.* **296**, 109990 (2020).

30. Song, S., Shi, Y., Liu, N. & Liu, F. Theoretical screening and experimental synthesis of ultrahigh-iodine capture covalent organic frameworks. *ACS Appl. Mater. Interfaces* **13**, 10513-10523 (2021).

31. Xie, Y., Pan, T., Lei, Q., Chen, C., Dong, X., Yuan, Y., Maksoud, W. A., Zhao, L., Cavallo, L., Pinnau, I. & Han, Y. Efficient and simultaneous capture of iodine and methyl iodide achieved by a covalent organic framework. *Nat. Commun.* **13**, 2878 (2022).

32. Yin, Z.-J., Xu, S.-Q., Zhan, T.-G., Qi, Q.-Y., Wu, Z.-Q. & Zhao, X. Ultrahigh volatile iodine uptake by hollow microspheres formed from a heteropore covalent organic framework. *Chem. Commun.* **53**, 7266-7269 (2017).

33. Zhang, M., Li, Y., Yuan, W., Guo, X., Bai, C., Zou, Y., Long, H., Qi, Y., Li, S., Tao, G., Xia, C. & Ma, L. Construction of flexible amine-linked covalent organic frameworks by catalysis and reduction of formic acid via the eschweiler-clarke reaction. *Angew. Chem. Int. Ed.* **60**, 12396-12405 (2021).

34. Zhao, Y., Liu, X., Li, Y., Xia, M., Xia, T., Sun, H., Sui, Z., Hu, X.-M. & Chen, Q. Ultra-stable fluorescent 2D covalent organic framework for rapid adsorption and selective detection of radioiodine. *Micropor. Mesopor. Mater.* **319**, 111046 (2021).

35. Xie, Y., Pan, T., Lei, Q., Chen, C., Dong, X., Yuan, Y., Shen, J., Cai, Y., Zhou, C., Pinnau, I. & Han, Y. Ionic functionalization of multivariate covalent organic frameworks to achieve an exceptionally high iodine-capture capacity. *Angew. Chem. Int. Ed.* **133**, 22606-22614 (2021).

36. An, S., Zhu, X., He, Y., Yang, L., Wang, H., Jin, S., Hu, J. & Liu, H. Porosity modulation in two-dimensional covalent organic frameworks leads to enhanced iodine adsorption performance. *Ind. Eng. Chem. Res.* **58**, 10495-10502 (2019).

37. Guo, X., Li, Y., Zhang, M., Cao, K., Tian, Y., Qi, Y., Li, S., Li, K., Yu, X. & Ma, L. Colyliform crystalline 2D covalent organic frameworks (COFs) with quasi-3D topologies for rapid I_2 adsorption. *Angew. Chem. Int. Ed.* **59**, 22697-22705 (2020).

38. He, L. W., Chen, L., Dong, X. L., Zhang, S. T., Zhang, M. X., Dai, X., Liu, X. J., Lin, P., Li, K. F., Chen, C. L., Pan, T. T., Ma, F. Y., Chen, J. C., Yuan, M. J., Zhang, Y. G., Chen, L., Zhou, R. H., Han, Y., Chai, Z. F. & Wang, S. A nitrogen-rich covalent organic framework for simultaneous dynamic capture of iodine and methyl iodide. *Chem* **7**, 699-714 (2021).

39. Wang, P., Xu, Q., Li, Z., Jiang, W., Jiang, Q. & Jiang, D. Exceptional iodine capture in 2D covalent organic frameworks. *Adv. Mater.* **30**, 1801991 (2018).

40. Jie, K., Zhou, Y., Sun, Q., Li, B., Zhao, R., Jiang, D. E., Guo, W., Chen, H., Yang, Z., Huang, F. & Dai, S. Mechanochemical synthesis of pillar[5]quinone derived multi-microporous organic polymers for radioactive organic iodide capture and storage. *Nat. Commun.* **11**, 1086 (2020).

41. Chen, G., Zhao, Q., Wang, Z., Jiang, M., Zhang, L., Duan, T. & Zhu, L. Pitch-based porous polymer beads for highly efficient iodine capture. *J. Hazard. Mater.* **434**, 128859-128859 (2022).

42. Zhang, H.-p., Gong, L.-l., Yin, M.-j., Xiong, X.-h., Zhang, Q.-y., Feng, X.-f., Luo, F., Carney, J. B. & Yue, Y. Efficient organic iodide capture by a mesoporous bimetallic-organic framework. *Cell Rep. Phys. Sci.* **3**, 100830 (2022).

43. Li, B., Dong, X., Wang, H., Ma, D., Tan, K., Jensen, S., Deibert, B. J., Butler, J., Cure, J., Shi, Z., Thonhauser, T., Chabal, Y. J., Han, Y. & Li, J. Capture of organic iodides from nuclear waste by metal-organic framework-based molecular traps. *Nat. Commun.* **8**, 485 (2017).

44. Chebbi, M., Azambre, B., Volkringer, C. & Loiseau, T. Dynamic sorption properties of metal-organic frameworks for the capture of methyl iodide. *Micropor. Mesopor. Mater.* **259**, 244-254 (2018).

45. Cha, G.-Y., Sivan, S. E., Lee, M., Oh, K.-R., Valekar, A. H., Kim, M.-K., Jung, H., Hong, D.-Y. & Hwang, Y. K. Ag-exchanged mesoporous chromium terephthalate with sulfonate for removing radioactive methyl iodide at extremely low concentrations in humid environments. *J. Hazard. Mater.* **417**, 125904 (2021).

46. Tung Cao Thanh, P., Docao, S., Hwang, I. C., Song, M. K., Choi, D. Y., Moon, D., Oleynikov, P. & Yoon, K. B. Capture of iodine and organic iodides using silica zeolites and the semiconductor behaviour of iodine in a silica zeolite. *Energy Environ. Sci.* **9**, 1050-1062 (2016).

47. Zhang, Y., He, L., Pan, T., Xie, J., Wu, F., Dong, X., Wang, X., Chen, L., Gong, S., Liu, W., Kang, L., Chen, J., Chen, L., Chen, L., Han, Y. & Wang, S. Superior iodine uptake capacity enabled by an open metal-sulfide framework composed of three types of active sites. *CCS Chem.* **0**, 1-9 (2022).

48. Zhang, Z., Dong, X., Yin, J., Li, Z. G., Li, X., Zhang, D., Pan, T., Lei, Q., Liu, X., Xie, Y., Shui, F., Li, J., Yi, M., Yuan, J., You, Z., Zhang, L., Chang, J., Zhang, H., Li, W., Fang, Q., Li, B., Bu, X. H. & Han, Y. Chemically stable guanidinium covalent organic framework for the efficient capture of low-concentration iodine at high temperatures. *J. Am. Chem. Soc.* **144**, 6821-6829 (2022).

49. Xie, L., Zheng, Z., Lin, Q., Zhou, H., Ji, X., Sessler, J. L. & Wang, H. Calix[4]pyrrole-based crosslinked polymer networks for highly effective iodine adsorption from water. *Angew. Chem. Int. Ed.* **61**, e202113724 (2021).

50. Yang, M., Qiu, F., ES, M. E.-S., Wang, W., Du, S., Su, K. & Yuan, D. Water-stable hydrazone-linked porous organic cages. *Chem. Sci.* **12**, 13307-13315 (2021).

51. An, D., Li, L., Zhang, Z., Asiri, A. M., Alamry, K. A. & Zhang, X. Amino-bridged covalent organic polycalix 4 arenes for ultra efficient adsorption of iodine in water. *Mater. Chem. Phys.* **239**, 122328 (2020).

52. Xu, X.-H., Li, Y.-X., Zhou, L., Liu, N. & Wu, Z.-Q. Precise fabrication of porous polymer frameworks using rigid polyisocyanides as building blocks: from structural regulation to efficient iodine capture. *Chem. Sci.* **13**, 1111-1118 (2022).

53. Wang, J., Li, Z., Wang, Y., Wei, C., Ai, K. & Lu, L. Hydrogen bond-mediated strong adsorbent-I_3^- interactions enable high-efficiency radioiodine capture. *Mater. Horiz.* **6**, 1517-1525 (2019).

54. Liu, Y. J., Sun, Y. F., Shen, S. H., Wang, S. T., Liu, Z. H., Fang, W. H., Wright, D. S. & Zhang, J. Water-stable porous Al_{24} Archimedean solids for removal of trace iodine. *Nat. Commun.* **13**, 6632 (2022).

55. Lin, Y., Jiang, X., Kim, S. T., Alahakoon, S. B., Hou, X., Zhang, Z., Thompson, C. M., Smaldone, R. A. & Ke, C. An elastic hydrogen-bonded cross-linked organic framework for effective iodine capture in water. *J. Am. Chem. Soc.* **139**, 7172-7175 (2017).

56. Zhang, M., Samanta, J., Atterberry, B., Staples, R., Rossini, A. J. & Ke, C. A Crosslinked ionic organic framework for efficient iodine and iodide remediation in water. *Angew. Chem. Int. Ed.* **61**, e202214189 (2022).

57. Li, B., Wang, B., Huang, X., Dai, L., Cui, L., Li, J., Jia, X. & Li, C. Terphen n arenes and quaterphen n arenes (n=3-6): One-pot synthesis, self-assembly into supramolecular gels, and iodine capture. *Angew. Chem. Int. Ed.* **58**, 3885-3889 (2019).

58. Zhao, Q., Chen, G., Wang, Z., Jiang, M., Lin, J., Zhang, L., Zhu, L. & Duan, T. Efficient removal and immobilization of radioactive iodide and iodate from aqueous solutions by bismuth-based composite beads. *Chem. Eng. J.* **426**, 131629 (2021).

59. Chen, R., Hu, T. & Li, Y. Stable nitrogen-containing covalent organic framework as porous adsorbent for effective iodine capture from water. *React. Funct. Polym.* **159**, 104806 (2021).

60. Becke, A. D. Density-functional exchange-energy approximation with correct asymptotic behavior. *Phys. Rev. A* **38**, 3098-3100 (1988).

61. Lee, C., Yang, W. & Parr, R. G. Development of the colle-salvetti correlation-energy formula into a functional of the electron density. *Phys. Rev. B* **37**, 785-789 (1988).

62. Becke, A. D. Density-functional thermochemistry. III. The role of exact exchange. *J. Chem. Phys.* **98**, 5648-5652 (1993).

63. Petersson, G. A., Bennett, A., Tensfeldt, T. G., Al-Laham, M. A., Shirley, W. A. & Mantzaris, J. A complete basis set model chemistry. I. The total energies of closed-shell atoms and hydrides of the first-row elements. *J. Chem. Phys.* **89**, 2193-2218 (1988).

64. Petersson, G. A. & Al-Laham, M. A. A complete basis set model chemistry. II. Open-shell systems and the total energies of the first-row atoms. *J. Chem. Phys.* **94**, 6081-6090 (1991).

65. Bergner, A., Dolg, M., Küchle, W., Stoll, H. & Preuß, H. Ab initio energy-adjusted pseudopotentials for elements of groups 13–17. *Mol. Phys.* **80**, 1431-1441 (2006).

66. Marenich, A. V., Cramer, C. J. & Truhlar, D. G. Universal solvation model based on solute electron density and on a continuum model of the solvent defined by the bulk dielectric constant and atomic surface tensions. *J. Chem. Phys. B* **113**, 6378-6396 (2009).

67. Lu, T. & Chen, Q. Shermo: A general code for calculating molecular thermochemistry properties. *Comput. Theor. Chem.* **1200**, 113249 (2021).

68. Frisch, M. J., Trucks, G. W., Schlegel, H. B., Scuseria, G. E., Robb, M. A., Cheeseman, J. R., Scalmani, G., Barone, V., Petersson, G. A., & et al., *Gaussian 16 Rev. B.01*, Wallingford, CT (2016).

69. Lu, T. & Chen, F. Quantitative analysis of molecular surface based on improved marching tetrahedra algorithm. *J. Mol. Graph. Model.* **38**, 314-323 (2012).

70. Lu, T. & Chen, F. Multiwfn: a multifunctional wavefunction analyzer. *J. Comput. Chem.* **33**, 580-592 (2012).
