# Supplementary material for: Engineering the pore environment of antiparallel stacked covalent organic frameworks for capture of iodine pollutants
Source: Nat Commun. 2024 Mar 26;15:2671. doi: 10.1038/s41467-024-46942-0 (PMC10965901; doi:10.1038/s41467-024-46942-0)
Supplement: Supplementary file 3 — Reporting Summary [file 41467_2024_46942_MOESM3_ESM.pdf]

nature portfolio

Corresponding author(s): Hui Yang, Xiangke Wang, Shengqian Ma

Last updated by author(s): Feb 22, 2024

Reporting Summary

Nature Portfolio wishes to improve the reproducibility of the work that we publish. This form provides structure for consistency and transparency in reporting. For further information on Nature Portfolio policies, see our Editorial Policies and the Editorial Policy Checklist.

Statistics

For all statistical analyses, confirm that the following items are present in the figure legend, table legend, main text, or Methods section.

n/a	Confirmed	
☐	☒	The exact sample size (*n*) for each experimental group/condition, given as a discrete number and unit of measurement
☐	☒	A statement on whether measurements were taken from distinct samples or whether the same sample was measured repeatedly
☒	☐	The statistical test(s) used AND whether they are one- or two-sided<br>*Only common tests should be described solely by name; describe more complex techniques in the Methods section.*
☐	☒	A description of all covariates tested
☐	☒	A description of any assumptions or corrections, such as tests of normality and adjustment for multiple comparisons
☒	☐	A full description of the statistical parameters including central tendency (e.g. means) or other basic estimates (e.g. regression coefficient) AND variation (e.g. standard deviation) or associated estimates of uncertainty (e.g. confidence intervals)
☒	☐	For null hypothesis testing, the test statistic (e.g. *F*, *t*, *r*) with confidence intervals, effect sizes, degrees of freedom and *P* value noted<br>*Give P values as exact values whenever suitable.*
☒	☐	For Bayesian analysis, information on the choice of priors and Markov chain Monte Carlo settings
☐	☒	For hierarchical and complex designs, identification of the appropriate level for tests and full reporting of outcomes
☒	☐	Estimates of effect sizes (e.g. Cohen's *d*, Pearson's *r*), indicating how they were calculated

Our web collection on statistics for biologists contains articles on many of the points above.

Software and code

Policy information about availability of computer code

Data collection	No software was used.
Data analysis	Gaussian 16 program suite was used for density functional theory (DFT) calculations. Materials Studio 8.0 was used for structural simulations. Total charge density and interlayer differential charge density was used for Vienna ab initio simulation package (VASP). Fit2D software was used for small and wide-angle X-ray scattering (SAXS/WAXS) data analysis.

For manuscripts utilizing custom algorithms or software that are central to the research but not yet described in published literature, software must be made available to editors and reviewers. We strongly encourage code deposition in a community repository (e.g. GitHub). See the Nature Portfolio guidelines for submitting code & software for further information.

Data

Policy information about availability of data

All manuscripts must include a data availability statement. This statement should provide the following information, where applicable:

- Accession codes, unique identifiers, or web links for publicly available datasets
- A description of any restrictions on data availability
- For clinical datasets or third party data, please ensure that the statement adheres to our policy

The authors declare that all the data supporting the findings of this study are available within the article (and Supplementary Information Files), or available from the corresponding author on reasonable request.

Human research participants

Policy information about studies involving human research participants and Sex and Gender in Research.

Reporting on sex and gender	n/a
Population characteristics	n/a
Recruitment	n/a
Ethics oversight	n/a

Note that full information on the approval of the study protocol must also be provided in the manuscript.

Field-specific reporting

Please select the one below that is the best fit for your research. If you are not sure, read the appropriate sections before making your selection.

☐ Life sciences ☐ Behavioural & social sciences ☒ Ecological, evolutionary & environmental sciences

For a reference copy of the document with all sections, see nature.com/documents/nr-reporting-summary-flat.pdf

Ecological, evolutionary & environmental sciences study design

All studies must disclose on these points even when the disclosure is negative.

Study description	We report robust covalent organic frameworks possessing unique antiparallel stacked structures (denoted as ACOF-1 and ACOF-1R), good radiation resistance, high chemical stability, and excellent selectivity towards I2, CH3I, and I3−. Our COFs thus meet the numerous technical challenges of removing I2, CH3I, and I3− under various conditions, allowing the fast dynamic capture of I2 vapor and CH3I from off-gas streams and I3− dissolved in contaminated water sources. No human participiants/human samples/cell lines/ antibodies/clinical data were used in the research.
Research sample	Covalent organic frameworks (COFs) represent an emerging class of organic adsorbents, which have been explored as adsorbents for iodine capture.
Sampling strategy	2,5-Dibutoxyterephthalohydrazide (DBTH, 99%) and 5,5',5''-(benzene-1,3,5-triyl)tripicolinaldehyde (BTPA, 98%) were purchased from Jilin Chinese Academy of Sciences-Yanshen Technology Co., Ltd. Ethanol (99.7%), Methanol (99.5%), 1,4-Dioxane (99%), Acetic acid (99.5%), N,N-Dimethylformamide (99.5%), 1,3,5-Trimethylbenzene (99%), Cyclohexane (99.7%), Methyl trifluoromethanesulfonate (97%), D311 macroporous weak alkaline acrylic anion exchange resin, Macropore adsorptive resin D101, D152 macroporous acrylic weak acid cation exchange resin, Amberlite IRA402, and Activated carbon were purchased from Shanghai Macklin Biochemical Co., Ltd (Shanghai, China). Ultrapure water was obtained from a Millipore system (18.25 MΩ·cm). All chemicals were sourced from commercial suppliers and used without further purification. The groundwater was collected in Mentougou, Beijing, China.
Data collection	COF characterization studies used the following techniques. Powder X-ray diffraction (PXRD) patterns were collected on a Rigaku SmartLab SE X-ray diffractometer equipped with a Cu Kα source. Fourier transform infrared spectra (FT-IR) were recorded on a SHIMADZU IRTracer-100. Scanning electron microscopy (SEM) images were recorded on TESCAN MIRA4 and TESCAN MIRA LMS Scanning Electron Microscopes. Transmission electron microscopy (TEM) images were obtained on a JEM-ARM200F electron microscope operating at 200 kV. High-resolution TEM images were obtained on a Thermo Fisher Scientific Titan Themis Z transmission electron microscope operating at 60 kV and equipped with a spherical aberration corrector. Solid-state 13C CP/MAS NMR spectra were recorded on a Bruker AVANCE III HD 600 WB spectrometer with a 4.0 mm MAS probe and spin rate of 10 kHz. X-ray photoelectron spectroscopy (XPS) analyses were performed using a Thermo Scientific ESCALAB 250Xi spectrometer, equipped with a monochromatic Al Kα X-ray source. Thermogravimetric analyses (TGA) were performed on TESCAN MIRA LMS under a flow of nitrogen by heating samples from room temperature to 800 °C at a rate of 10 °C/min. BET surface areas were determined from N2 adsorption/desorption isotherms collected at 77 K on a Micromeritics ASAP 2020 volumetric adsorption analyzer. Elemental analysis was performed on an Elemental Vario EL cube. Raman spectra were obtained from powder samples on a Jobin Yvon HR-800 Raman spectrometer equipped with a cobalt samba single-mode 514 nm diode laser. UV-vis spectra were recorded on a SHIMADZU UV-2700 UV-Vis spectrophotometer. UV-vis single point data were recorded on a Techcomp S1020 UV-Vis spectrophotometer. Dynamic I2 and CH3I adsorption experiments were conducted on a custom-built breakthrough system. CH3I in the effluent from the adsorbent column was quantified using a gas chromatograph (Shimadzu GC2030) equipped with a flame ionization detector and GC column (SH-1, 0.25 mm×0.25 μm×30 m). Small and wide-angle X-ray scattering patterns were performed using a Xenocs Xeuss 3.0 equipped with a Cu microfocus sealed tube (30 W/30 μm) and generated at 50 kV and 0.6 mA. Dried sample powders were encapsulated with amorphous tape, then fixed to the sample holder using another piece of tape. The scattering patterns were recorded using Eiger2R 1M detectors for the SAXS and WAXS measurement, $q = 4\pi \sin\theta/\lambda$. Fit2D software was used for the data reduction with normalized circle gathering.<br><br>Density functional theory (DFT) calculations<br>Total charge density and interlayer differential charge density calculations<br>Density functional theory (DFT) calculations were carried out in the Vienna ab initio simulation package (VASP) based on the plane-

wave basis sets with the projector augmented-wave method. The exchange-correlation potential was treated by using a generalized gradient approximation (GGA) with the Perdew-Burke-Ernzerhof (PBE) parametrization. Van der Waals interactions were considered at the vdW-DF level with the nonlocal vdW-DF2 functional. The energy cutoff was set to be 500 eV. The Brillouin-zone integration was sampled with a Γ-centered Monkhorst-Pack mesh of 1 × 1 × 3 (1 × 1 × 1) for antiparallel and eclipsed cells, respectively. The structures were fully relaxed until the maximum force on each atom was less than 0.01 eV/Å, and the energy convergent standard was 10-5 eV.

A fragment of ACOF-1, ACOF-1R (CF3SO3−), I2, I3−, I5− and the structures for adsorbed iodine species on COFs were all optimized under the framework of the density of functional theory (DFT) with B3LYP functional, using a DFT-D3 dispersion correction method and basis set of 6-31g(d)+SDD. Here, the SDD effective core potential was used to describe the atomic orbitals and relativistic effects of iodine atoms. The reactants, products, and transition state of the methylation reaction of pyridine were also calculated as above. Vibrational frequency analyses were carried out for the optimized structures using the same calculation method. In order to describe solvation effects, the SMD (Solvation Model Based on Density)66 implicit solvent model was used in the reaction calculations. The thermodynamic correction terms and Gibbs free energy of the structures at 298.15K were then obtained using the Shermo program. In order to obtain the electron energy with higher accuracy, single point calculations for the optimized structures with B3LYP functional and 6-311+G(d,p) & SDD basis set were performed. Finally, the single point energy was added to the free energy correction calculated before obtaining the Gibbs free energy. All these DFT calculations above were performed using Gaussian 16 program suite.

The adsorption energy of I2/hydrazine sites, I2/pyridine sites, I3−/pyridine sites, and I5−/pyridine sites were calculated from the formula:
"E" ("adsorb")"=E" ("A+B")"-E" ("A")"-E" ("B")

where E(A) and E(B) are the free energy of isolated molecules, E(A+B) is the total energy of the adsorption complex structures.

The electrostatic surface potentials (ESP) were calculated using the Multiwfn program and then rendered using the Gauss View 6.0.

H.Y., X.W., and S.M. conceived and designed the research. Y.X., Q.R., X.L., and M.H. performed the synthesis and characterization. Y.X., Q.R., F.M., Y.W., and S.W. carried out the adsorption experiments. Y.X., X.L. and Z.C. performed and analyzed the TEM and DFT calculations. H.Y., X.W., G.I.N.W., and S.M. wrote the manuscript. All authors contributed to the discussion, and gave approval to the final version of the manuscript.

Timing and spatial scale	The research work was conducted from April 2021 to November 2023.
Data exclusions	No data exclusions.
Reproducibility	All attempts to repeat the experimental findings were successful (at least repeated three times).
Randomization	Data obtained under the same testing conditions were allocated into the same group.
Blinding	No blinding was used. This work is not a clinical research (or related research) study.

Did the study involve field work? ☐ Yes ☒ No

Reporting for specific materials, systems and methods

We require information from authors about some types of materials, experimental systems and methods used in many studies. Here, indicate whether each material, system or method listed is relevant to your study. If you are not sure if a list item applies to your research, read the appropriate section before selecting a response.

Materials & experimental systems

n/a	Involved in the study
☒	☐ Antibodies
☒	☐ Eukaryotic cell lines
☒	☐ Palaeontology and archaeology
☒	☐ Animals and other organisms
☒	☐ Clinical data
☒	☐ Dual use research of concern

Methods

n/a	Involved in the study
☒	☐ ChIP-seq
☒	☐ Flow cytometry
☒	☐ MRI-based neuroimaging
